# Towards a second generation of ‘social media metrics’: Characterizing Twitter communities of attention around science

**DOI:** 10.1371/journal.pone.0216408

**Published:** 2019-05-22

**Authors:** Adrián A. Díaz-Faes, Timothy D. Bowman, Rodrigo Costas

**Affiliations:** 1 INGENIO (CSIC-UPV), Universitat Politécnica de València, Valencia, Spain; 2 School of Information Sciences, Wayne State University, Detroit, Michigan, United States of America; 3 Centre for Science and Technology Studies (CWTS), Leiden University, Leiden, The Netherlands; 4 Centre for Research on Evaluation, Science and Technology (CREST), Stellenbosch University, Stellenbosch, South Africa; Max Planck Society, GERMANY

## Abstract

‘Social media metrics’ are bursting into science studies as emerging new measures of impact related to scholarly activities. However, their meaning and scope as scholarly metrics is still far from being grasped. This research seeks to shift focus from the consideration of social media metrics around science as mere indicators confined to the analysis of the use and visibility of publications on social media to their consideration as metrics of interaction and circulation of scientific knowledge across different communities of attention, and particularly as metrics that can also be used to characterize these communities. Although recent research efforts have proposed tentative typologies of social media users, no study has empirically examined the full range of Twitter user’s behavior within Twitter and disclosed the latent dimensions in which activity on Twitter around science can be classified. To do so, we draw on the overall activity of social media users on Twitter interacting with research objects collected from the Altmetic.com database. Data from over 1.3 million unique users, accounting for over 14 million tweets to scientific publications, is analyzed. Based on an exploratory and confirmatory factor analysis, four latent dimensions are identified: ‘Science Engagement’, ‘Social Media Capital’, ‘Social Media Activity’ and ‘Science Focus’. Evidence on the predominant type of users by each of the four dimensions is provided by means of VOSviewer term maps of Twitter profile descriptions. This research breaks new ground for the systematic analysis and characterization of social media users’ activity around science.

## Introduction

Events related to research objects in online environments, commonly referred to as ‘altmetrics’ and more specifically as ‘social media metrics’ [1] given their social media component, are increasingly being included in quantitative science studies as appealing new measures of social media interactions and knowledge dissemination. Their potential as traces of previously invisible features of scholarly communication were set forth in the ‘Altmetrics manifesto’ by Priem et al. [2]. Social media metrics bring to the foreground a family of new indicators to gauge the dissemination and reception of research outside the traditional academic circles by providing recent data on various events including shares, users, readers, downloads, comments, or recommendations in social media sites such as Twitter, Facebook, News media, Mendeley, and blogs. Despite its limitations, the analysis of scientific activity through scientometric methods has a long and well-established tradition that dates back to the early 20^th^ century [3–5]. With the creation and development of bibliographic databases such as Web of Science (WoS) or Scopus, citation, authorship, acknowledgments, and productivity indicators have become valuable tools for digging into the dynamics of scientific activity and the (co)creation of knowledge, as well as for research assessment processes [6]. In contrast, the scope and meanings of social media metrics are still far from being grasped [7]. Current research is also pointing to data quality issues as a major challenge for altmetrics and social media metrics development [8]. Transparency on how data providers collect, aggregate, and compute metrics arises as a fundamental issue in the quest for reliability, reproducibility, and validity of social media metrics [9]. In fact, the current state of social media metrics can be equated to that which was underwent by bibliometrics in the 1970s [10].

### Approaching societal impact from social media metrics

The emergence and development of social media metrics parallels the increasing policy demands for moving beyond the hegemonic assessment model, which is based on productivity and citations, to analyzing the societal impact of research [11], as well as responding to initiatives that advocate for better practices in research evaluation (such as the DORA declaration). This rationale relies on the idea that scientific research should also drive social change and respond to societal demands [12, 13]. From this standpoint, the societal impact of research can be seen as being as meaningful as the scientific impact.

The consideration of societal impact from the prism of social media metrics implies shifting the focus from research entities (outputs, citations, researchers, institutions) to the processes of *interaction* and the circulation of scientific knowledge across broader online communities (tweeters, Facebook, readership, etc.). Haustein, Bowman, and Costas [14] indicate that the traces left by the interaction of users with research objects provide metrics on the *access* (viewing, downloading or saving), *appraisal* (mentioning, commenting or reviewing) and *application* (using, adapting or transforming) of said objects. This interactive perspective binds together forms of symbolic capital from two very different realms: science and social media [15–16].

The interest of social media metrics as potential traces of societal impact particularly stands out in domains such as *biomedical and health sciences*, where research may lead to changes that improve human well-being; and in *humanities*, which deals with human behavior and social relations between people and organizations [17]. On one hand, the advances of biomedical research and the sharing of these advances with society seems particularly relevant given its complexity [18], influence on human quality of life, the large amount of money invested by public research organizations, and the importance of social awareness for achieving behavioral changes. On the other hand, societal impact in humanities is also crucial as research in this domain provides new approaches, reflections, and criticism on human experience and their modes of expression [19, 20]. Not surprisingly, research outputs from these two domains demonstrate the highest presence on social media platforms [21].

### Twitter as a strong source of social media metrics

The microblogging platform Twitter stands as the most appealing context in which to analyze the processes of interaction and the circulation of scientific knowledge, as it is used by a wide-ranging number of academic and non-academic users [7, 22]. These users discuss issues that span from local to global topics and from professional to personal interests. Social media activity revolving around research objects continues to grow [23] and Twitter is one of the social media platforms with the largest activity [24], only outperformed by Mendeley [25]. Haustein, Costas, and Larivière [26] found that the presence of research objects in other social media platforms (excluding Twitter) is very low (less than 5% of papers are shared or mentioned on Facebook, blogs, Google+, and mainstream media). These authors demonstrated that Twitter is by far the most widespread platform for open social media dissemination with around 21% of papers receiving at least one tweet.

### Towards a second generation of social media metrics

Twitter is an important source of social media metrics, sometimes specifically referred to as ‘scholarly Twitter metrics’ [27]. However, previous studies on the content analysis of tweets linking to papers pointed to the existence of scarce original content and engagement with research objects [28], as well as tweets containing mostly neutral sentiments [29] expressed by tweeters. This lack of content engagement of tweeters with research objects reinforces the idea that the analysis of interactions of social media users with scholarly outputs is a preferable focal point in what Robinson-García, van Leeuwen, and Rafols [11] termed as “interaction approaches” (building on previous concepts such as “productive interactions” [17] for societal impact analysis). Similarly, Haustein [27] suggested that “Twitter is less about what people tweet rather how they are connected”, highlighting that “how, when, and by whom” research is tweeted is important “in order to understand what tweets to scholarly documents measure”. This more interactive perspective can also fit in the framework set out by Haustein, Bowman, and Costas [14], taking interactions as forms of lower engagement, where metrics are based on these interactions made by different social media users with research objects.

From a more general perspective, and following the suggestion by Costas [16], it can be argued that the analysis, understanding, and modelling of the interactions between *social media entities* (here including not only social media users [e.g. tweeters, Facebook users, Mendeley users, etc.], but also their social media activities [e.g. tweets, retweets, posts, likes, comments, etc.]) and *scholarly entities* (not being restricted just to scholarly outputs [e.g. publications, datasets, etc.], but also including scholarly agents such as scholars, research organizations, funders, journals, etc.) is what would comprise the core of the *Social Media Studies of Science*. Thus, the examination and characterization of social media users interacting with research objects gains a renewed importance in understanding the meaning of these interactions.

Considering all of the above and from a conceptual standpoint, two different *generations* of social media metrics can be proposed according to the indicators scope and integration within overall social media activity.

- *Primary social media metrics*. These are metrics of the use and visibility of publications on social media (e.g. tweets and Facebook counts, Mendeley readers, etc.). This first generation can be deemed as the more *traditional* social media metrics, which accounts for the quantification of mentions of research objects in social media (e.g. the number of tweets of a publication, the number of Facebook posts, etc.), including here both scholarly outputs (i.e. publications, datasets, patents etc.) as well as scholarly agents [14] (e.g. research groups, individual scholars, journals, research organizations, etc.). In other words, primary social media metrics are research objects-focused.
- *Secondary social media metrics*. These comprise metrics about the social media users and their online activities, including both their overall activity on social media (e.g. overall tweeting activities, likes, followers, etc.) as well as regarding their specific interactions with research objects (e.g. number of tweets given to papers, mentions of scholars in their tweets, etc.). In other words, secondary social media metrics are mainly focused on social media-objects (including both social media agents–e.g. tweeters, Facebook users, Mendeley users, etc.; and social media objects–e.g., tweets, retweets, likes, wall posts, etc.).

This distinction between primary and secondary social media metrics is sustained on the idea that so far social media metrics research has had a strong focus on the characterization of research objects by their reception in social media. Nevertheless, there have not been many attempts to develop metrics and analytics focused on characterizing those social media actors and their activities regarding their interactions with science and their social media activities overall. Thus, we propose a relatively novel approach in which the social media object becomes the focal point, over the more traditional approach in which the research object is the focal point. Given this fundamental change in perspective, we argue that distinguishing them as a *secondary* is relevant as they stem from primary social media metrics but represent an important change in the focus and potential interpretability and applicability of the metrics. For example, primary social media metrics can be calculated to capture some form of impact of a University’s publications on Twitter, Facebook, policy documents, etc. However, someone could also study the activity of the University’s Twitter (or Facebook) accounts, thus being more interested in the followers, followees, number of tweets sent, number of tweets to papers, number of retweets received, etc. by the University’s Twitter account. We argue that this second (secondary) form of social media metrics is different from the first one (primary), and we believe that this differentiation may help to distinguish them more efficiently. In the conceptualization of these *secondary social media metrics* (i.e. characterizing social media user interactions with research objects within the full range of activities on social media platforms) it is of particular importance that the characterization of the activities of these users be considered not only with regards to their interactions with scholarly entities, but also with their overall interactions within the different social media environments in which the events take place. The study of how social media users interact with research objects will help to pave the way to further unravel the mechanisms by which academic and, especially, non-academic actors interplay with scientific outputs and scholarly entities.

### Understanding Twitter users and their interactions with science

Several attempts have been made so far to classify users in the social media realm based on their behavior [1]. Brandtzæg [30] proposed a tentative unified Media User Typology, which categorized users based on their frequency and variety of use and content preferences. Kwak et al. [31] performed the first study on the entire Twittersphere analyzing 41.7 million user profiles using a network analysis lens to put forth the following/followees model. The authors ranked users by their number of followers and retweets, and found these two rankings to be different indicating “a gap in influence inferred from the number of followers and from the popularity of one’s tweets” [31] (p. 600). More specifically Altmetric.com classifies Twitter users as *researchers*, *science communicators*, *practitioners*, and *general public* based on keywords in profile descriptions, journals tweeted, and follower lists. Haustein & Costas [32] explored the potential of using Twitter account descriptions to typify users. Both approaches demonstrate certain overlap among categories and rely heavily on profile descriptions. A tentative classification of users based on their tweeting behavior was performed by Haustein, Bowman, and Costas [15], who based categories on two indicators—exposure (number of followers) and engagement (dissimilarity between tweet and paper title)—and distinguished four types of users: brokers [high engagement and exposure]; broadcasters [high exposure but low engagement]; orators [high engagement but low exposure]; and mumblers [low exposure and engagement].

Nevertheless, no study has previously identified the latent dimensions on which a social media user’s behavior relates to science communication; neither founded on the overall activity on Twitter nor including the specific interactions users have with research objects. By disclosing these underlying dimensions, different communities of users communicating science might be handily identified according to certain characteristics of their tweeting behavior.

The primary aim of this paper is to identify and conceptualize dimensions of social media metrics useful to characterize Twitter communities interacting with science. More specifically, the aims of the authors are twofold. The first aim is to propose a second generation of Twitter metrics able to outline the general activities of Twitter communities in their Twitter interactions with scientific outputs. Based on this second generation of Twitter metrics, the second aim is to set the groundwork for the potential identification of different types of Twitter users shaped by their forms of interaction with research objects and in their overall activities on Twitter.

## Material and methods

### Twitter metrics data processing

Altmetric.com shared their full dataset (v 10/02/2017) with the authors during the month of October, 2017. Approximately 8.1 million research objects with their different ‘altmetric’ events were parsed and stored in a local relational database. With regards to Twitter, each tweet was stored in a table relating back to the main Altmetric.com record for the scholarly output. The data revealed that there was a total of over 4 million Twitter users in the Altmetric.com database. Altmetric.com does not, however, share all of the user information of the tweeters that is available through the Twitter API. This missing information includes data such as the total number of tweets of the user, the date of creation of the account, the number of followees, and the number of likes. None of these indicators are currently included in the Altmetric.com data dump. Therefore, a PHP script was written to query the Twitter Search API using the unique tweet ID captured by Altmetric.com in order to obtain a complete record of the tweeter profile information. The script was executed on a LAMP server at the beginning of the month of February, 2018 and the data was collected, parsed, and stored in a relational database table.

### Sample

The final profile data of Twitter users retrieved was of 3,580,727 unique profiles; each user shared at least one ‘tweet’ mentioning a scholarly output between August 2011 and October 2017. To ensure data accuracy, the population was constrained to those users (n = 1,448,867) who had a complete overlap of their Twitter activity with the coverage period of Altmetric.com, thus users that created their Twitter account before August 2011 were excluded. In addition, the influence of extremely low active profiles was diminished by removing those users belonging to the bottom 5% least active on Twitter overall; only users with more than a total of 15 tweets over the six-year period were included. The final dataset is comprised of 1,340,695 users accounting for over 14.6 million tweets to scholarly outputs.

### Metrics

Based on the amount of information and data collected, a number of new metrics were developed in order to characterize the behavior of users on Twitter (all these metrics can be considered as secondary social media metrics, since they have been computed at the user level). In this work, the authors focused on metrics that were relatively simple and feasible given the data at hand. Other metrics would be possible to determine (e.g. number of comments received by a tweet to a paper, number of tweets sent mentioning other tweeters, replies, etc.), but their calculation would be more complicated and uncertain. Abbreviations are provided in square brackets.

- Number of (re)tweets linking to scientific publications [tws]: absolute number of tweets and retweets to scholarly outputs made by each user.
- Number of original tweets (i.e. not retweets) linking to scientific publications [otw].
- Number of distinct publications (re)tweeted [p tw]: total actual number of distinct scholarly outputs tweeted.
- Number of (re)tweets containing hashtags [tws hash].
- Average length of the titles of the papers tweeted [avg title length].
- Average time between the publication of the paper (as in Altmetric.com) and the tweet of the tweeter [avg days to tweet pub].
- Number of tweets overall [tweets]: this is the number of tweets recorded in the Twitter profile of the tweeter.
- Number of followers [followers]: this is the number of followers recorded in the Twitter profile of the tweeter.
- Number of followees [followees]: this is the number of followees recorded in the Twitter profile of the tweeter.
- Number of likes given [likes given]: this is the number of likes given by the user recorded in the Twitter profile of the tweeter.
- Number of lists in which users are listed [listed count]: this is the number of lists recorded in the Twitter profile of the tweeter.
- Share of tweets to papers [ptws to paper]: share of the total number of tweets that users have made to scholarly outputs.

### Data analysis

For data analysis, the authors used three analytical approaches described below. First, an Exploratory Factor Analysis (EFA) was conducted using Principal Component Analysis (PCA) for factor extraction. EFA aims to study the underlying latent structure of correlations among a set of variables. In this work, EFA allows grouping metrics that measure similar characteristics into dimensions based on the interrelations among them. Seeing that some variables differ in scale, the analysis is run based on the correlation matrix (i.e. standardized covariances). As some metrics, such as those referring to the interaction with scholarly outputs (e.g. *p tw*, *tws hash*, *ptws to papers*), stem directly from the number of tweets, the authors did not want to force factors to be orthogonal (i.e. to be independent). Accordingly, an oblimin oblique rotation was performed such that the number of metrics with high loadings was minimized making the factor easily interpretable [33]. By identifying latent factors that represent different aspects revolving around scholarly papers, a fine-grained classification of Twitter metrics can be proposed. Second, to provide a more robust basis of the dimensions of Twitter metrics around science, the adequacy of the metrics as indicators of the latent constructs was evaluated by means of a Confirmatory Factor Analysis (CFA). CFA differs from EFA in that the underlying factors are specified a priori and verified empirically rather than derived from the data [34]. Several goodness-of-fit indices were assessed to estimate how well the specified model accounts for the data [35]. Third, to characterize the predominant type of users for each of the four dimensions, several distance-based maps based on the Twitter descriptions of the users were constructed using VOSviewer [36]. This technique uses a method called visualization of similarities that, under certain conditions, is equivalent to a Sammon’s MDS [37].

## Results

### Descriptive statistics

Table 1 presents the descriptive statistics used in the factorial model. An extremely high skewness regarding Twitter users’ activities is quite apparent when comparing mean and median, as well their dispersion measures. This huge variability (e.g. *followers*: mean = 1,099.19, SD = 19,458.25; median = 170, IQR = 524–54 = 470) reflects that there is no one-size-fits-all user’s behavioral pattern. For instance, the *ptws to papers* is on average 1.62% and on median 0.20% by user, which implies that interacting with scholarly outputs represents, on average, a very small share of a user’s activity on Twitter.

**Table 1:** Descriptive statistics for Twitter metrics.

Variable	Mean	S.D.	Min	Max	P25	Median	P75
**tws**	10.96	196.56	1	89,998	1	1	4
**otw**	5.35	186.36	0	89,998	0	0	1
**tws hash**	3.65	93.92	0	65,577	0	0	1
**p tw**	9.22	133.05	1	59,300	1	1	3
**avg title length**	74.17	31.47	0	6,959	54	72	91
**avg days to tweet pub**	560.76	1653.63	0	42,933	6	56	375
**tweets**	7,429.55	26388.99	16	2,934,861	261	1,112	4404
**followers**	1,099.19	19458.25	0	12,915,964	54	170	524
**followees**	792.57	4184.79	0	2,232,164	111	284	721
**likes given**	4,598.32	15894.15	0	990,477	56	410	2302
**listed count**	27.97	132.42	0	20,827	1	5	19
**pwts to paper**	1.62%	5.20%	0.00%	100.00%	0.04%	0.20%	1.00%

The Spearman’s Rho correlations showed in Table 2 demonstrates a basic insight into the pattern of correlations among all the Twitter metrics. Indicators linking activity on Twitter related to the diffusion of scholarly outputs present high positive correlations between them (*tws*, *otw*, *tws hash*, and *ptw*). Predictably, the overall activity on Twitter (*tweets*) is relatively high and positively correlated with the number of *followers*, and especially, *likes given* (ρ = 0.684). Likewise, there is high negative correlation between *tweets* and *ptws to papers* (ρ = -0.854) and, to a lesser extent, between *ptws to papers* and *likes given* (ρ = -0.593), indicating that users who are very active on Twitter do not focus their tweeting activity primarily on sharing scholarly papers. In other words, this suggests that highly active users use this social media platform for a larger number of purposes other than tweeting research objects.

**Table 2:** Spearman’s rho correlations between Twitter metrics.

		1	2	3	4	5	6	7	8	9
1	**tws**	1.000								
2	**otw**	0.499	1.000							
3	**tws hash**	0.592	0.271	1.000						
4	**p tw**	0.978	0.478	0.585	1.000					
5	**avg title length**	0.128	0.035	0.109	0.128	1.000				
6	**avg days to tweet pub**	0.245	0.176	0.151	0.242	0.107	1.000			
7	**tweets**	0.114	-0.071	0.049	0.113	-0.055	0.128	1.000		
8	**followers**	0.206	0.111	0.183	0.205	-0.003	0.094	0.616	1.000	
9	**followees**	0.107	-0.021	0.116	0.109	-0.018	0.054	0.514	0.690	1.000
10	**likes given**	0.067	-0.170	0.038	0.073	-0.029	0.085	0.684	0.469	0.483
11	**listed count**	0.262	0.131	0.264	0.263	-0.035	0.070	0.566	0.733	0.557
12	**pwts to papers**	0.355	0.339	0.271	0.350	0.117	0.001	-0.854	-0.453	-0.416
		10	11	12						
10	**likes given**	1.000								
11	**listed count**	0.391	1.000	
12	**pwts to papers**	-0.593	-0.379	1.000

### Dimensions of Twitter activity around science

An EFA was applied to disentangle the underlying latent structure on which Twitter users’ metrics revolve. Results are displayed in Table 3. Four factors were retained based on the eigenvalues > 1, which is known as Kaiser’s criterion [38], accounting for over 62% of the total variance. The inspection of factor loadings reveals the extent to which each of the Twitter metric contributes to the meaning of each of the factors. Both a Kaiser-Meyer-Olkin measure of sampling adequacy (KMO = 0.734) and a Bartlett’s test of sphericity (p = 0.000) indicate that the data are sufficiently suitable for latent structure detection.

**Table 3:** EFA of communities of users around science. Factor loadings and correlation among factors.

	Science Engagement	Social Media Activity	Social Media Capital	Science Focus
tws	**0.966**	0.009	0.019	0.084
otw	**0.946**	-0.008	0.006	0.065
p tw	**0.889**	0.006	0.020	0.115
tws hash	**0.776**	0.015	0.022	0.033
tweets	0.032	**0.836**	0.258	-0.077
likes given	-0.004	**0.811**	0.064	-0.089
followers	0.003	-0.019	**0.785**	0.013
followees	0.001	0.161	**0.727**	-0.014
listed count	0.044	0.412	**0.613**	-0.030
avg title length	-0.006	0.048	-0.051	**0.754**
% of tweets to papers	0.249	-0.186	-0.030	**0.546**
avg days to tweet pub	0.011	0.060	-0.041	**-0.490**
**Correlations among factors (Spearman’s Rho)**				
Science Engagement	1.000			
Social Media Activity	-0.433	1.000		
Social Media Capital	0.175	0.157	1.000	
Science Focus	-0.363	0.062	-0.254	1.000

Note: PCA used as extraction method. Oblimin oblique rotation (structure matrix). Factor loadings roughly > = 0.500 are printed in bold

Factor 1 accounted for 27.4% of the variance comprising four Twitter metrics *(tws*, *otws*, *p tws* and *tws hash*), which relates to what can be categorized as ‘Science Engagement’ because it reflects to what extent sharing science-related material characterizes users’ Twitter activity. Factor 2 explained 15.7% of the total variance and grouped two indicators (*tweets*, *likes given*), which can be categorized as ‘Social Media Activity’ as it relates to what users share and what they pay attention to. Factor 3 accounted for 10% of the variance and grouped three indicators (*followers*, *followees*, *and listed count*), which mirror the ‘Social Media Capital’ attained by users. It is worth noting here that *listed count* also shows an appreciable loading on the ‘Social Media Activity’ dimension. Although the prominent feature of lists is bringing together users with some kind of proximity (e.g. cognitive, personal), this metric also reflects that the more active a user is on Twitter, the more likely it is that they create or join other users’ lists. Apart from this, dimensions are fairly robust since there are no other cross-loadings (e.g. metrics that load ≥ 0.32 on two or more factors) [39]. Lastly, Factor 4 explained 8.9% of the total variance in the Twitter metrics grouping three items (*average title length*, *share of tweets to papers*, *avg days to tweet pub*), which reflect what can be categorized as ‘Science Focus’.

Furthermore, correlations between the four dimensions are also shown in the bottom of Table 3, which provide additional evidence regarding the underlying latent structure of interactions among users in the social media realm. Moderate negative correlations are found between ‘Social Media Activity’ and ‘Science Engagement’ (ρ = -0.433) and between ‘Social Media Activity’ and ‘Science Focus’ (ρ = -0.363). As aforementioned, this finding is not striking since ‘Social Engagement’, and to a lesser extent, ‘Science Focus’ metrics stem directly from the number of tweets (e.g. *tws*, *p tw*, *ptws to papers*).

To assess the validity of the four latent dimensions found through EFA, a CFA was performed. CFA tests if the hypothesized model is consistent with the actual data. Rather than making a dichotomous decision on the validity of the model, the CFA is intended to explore if the structure model gives a reasonable fit given the interdependence of several metrics from the number of tweets and the fact that interacting with scholarly outputs accounts for a very small share of users’ behavior on Twitter. Errors were included in the model along with some correlations among them based on large modification indices.

Goodness-of-fit indices are provided in Table 4 to evaluate if the specified model acceptably approximates the observed data. It is generally recommended to examine several indices simultaneously when model fit is assessed, since not all indices work equally under different conditions [34]. The result from the *χ*^*2*^ test is significant (p = 0.0008), which would suggest that the model does not fit the data. However, it is well-known that this test is very sensitive to the sample size, and significant results can be found despite actual differences being negligible [40]. Absolute and incremental indices attempt to adjust for the effect of sample size. Absolute indices estimate how well the conceptual model reproduces the sample data, whereas incremental indices assess the proportion of improvement in fit between the conceptual model and a more restricted model [41]. These indices must be considered to determine whether the model fit is acceptable or not [35]. Hu and Bentler [41] suggest the following cut-off values as good model fit: ≤ 0.08 for SRMR, ≤ 0.06 for RMSEA and ≥ 0.95 for incremental indices (CFI, TLI and IFI). As shown in Table 4, all values are respectively above/below the expected values, so it can be concluded that the model fit of our four dimensions of Twitter metrics is acceptable.

**Table 4:** Goodness-of-fit indices for the CFA.

Indices		Expected Value	Resultant Value
**Absolute fit**	*χ*^*2*^	< 0.05	0.000
	GFI	> 0.90	0.994
	SRMR	≤ 0.08	0.017
	RMSEA	< 0.06	0.029
**Incremental fit**	CFI		0.993
	TLI	≥ 0.95	0.989
	IFI		0.993

Note: Goodness-of-Fit Index (GFI), Standardized Root Mean Squared Residual (SRMSR), Root Mean Squared Error of Approximation (RMSEA), Comparative Fix Index (CFI), Tucker-Lewis Index (TLI), Incremental Fit Index (IFI).

### Communities of attention around science

**Fig 1:**
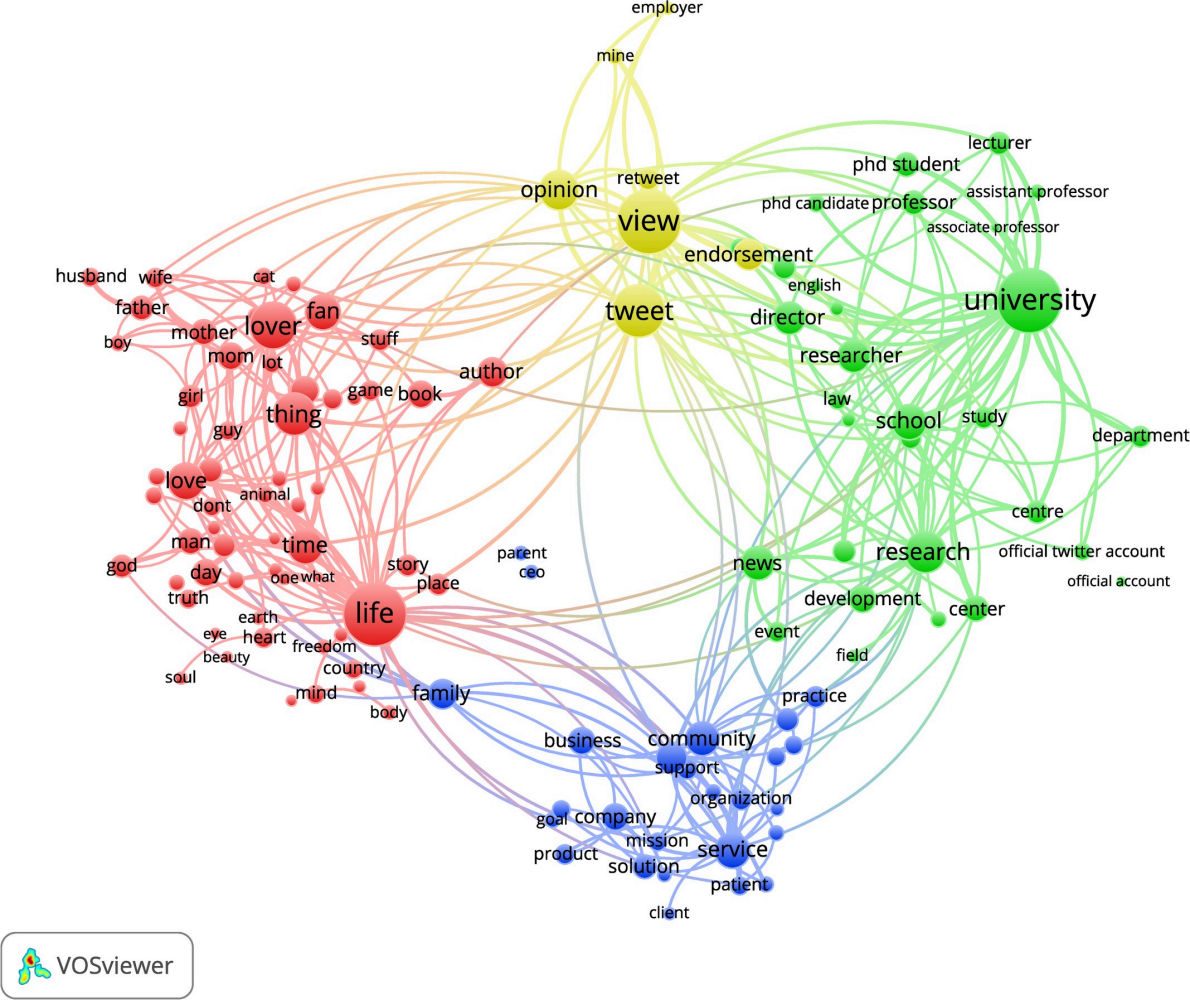
VOSviewer map of factor scores and profile descriptions.

To reveal the predominant type of users using the four latent dimensions, we draw on the user’s profile description. To do so, profile descriptions from 200,000 random Twitter accounts (from the 1,340,695 tweeters finally analysed) and factor scores for each dimension were used. Terms in the VOSviewer maps are placed in such a way that the distance between any pair of terms reflects their similarity in terms of co-occurrence. In other words, the proximity between terms indicates that the profiles are characterized by the same terms and represent a particular community of tweeters. The size of the nodes reflects the frequency of words and the thickness of the edges relate to how often the words appear together. A threshold of 200 occurrences was set for the terms, resulting in a total of 211 terms. For the sake of visualization, those terms accounting for over 60% of the occurrences were finally retained (a total of 127 terms). Fig 1 shows a terms map for the four dimensions where the main textual patterns of tweeters are brought to the foreground. We found four clearly defined clusters of users:

- Cluster 1 (red cluster) is formed by words such as ‘mother’, ‘lover’, ‘love’, ‘life’, ‘fan’, ‘husband’ or ‘soul,’ which reveal tweeters who set forth their private and personal life in their profile (*personal description*).
- Cluster 2 (yellow cluster) is comprised by terms that underline the expression of a user’s own opinions and views. Few words concentrate most of occurrences: ‘view’, ‘opinion’, ‘tweet’, ‘endorsement’ and ‘mine’ (*opinions and own position*).
- Cluster 3 (green cluster) includes words that belong to the academic and scientific community: ‘university’, ‘department’, ‘researcher’, ‘development’, ‘director’, ‘lecturer’ or ‘PhD student’ (*academic role*).
- Cluster 4 (blue cluster) is formed by words that refer to the professional role of tweeters, but in this case seems to be more connected with firms and professional practice. It includes words such as ‘organization’, ‘business’, ‘community’, ‘practice’, ‘service’, ‘client’ or ‘solution’ (*business and practice role*).

**Fig 2:**
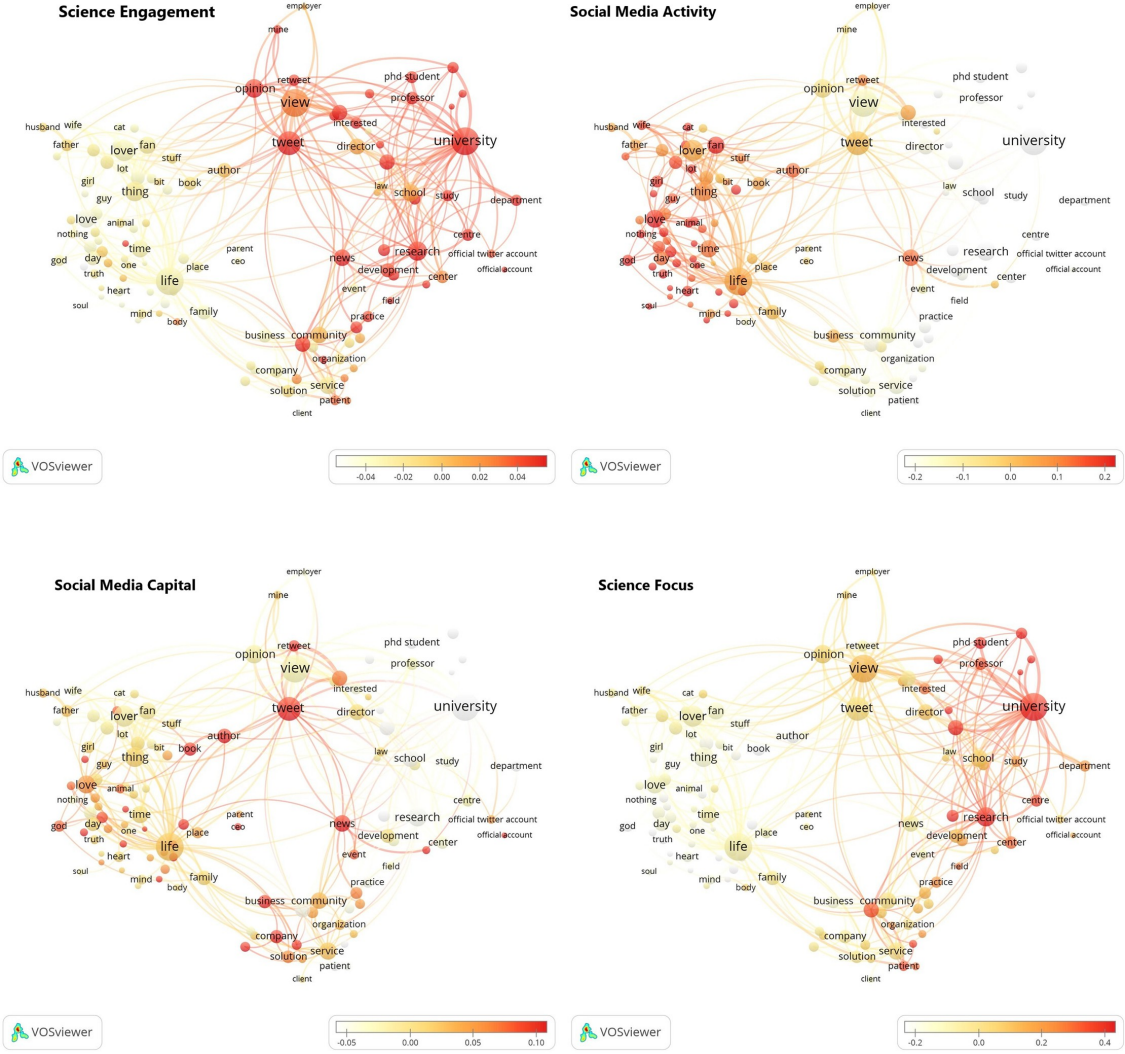
VOSviewer maps of profile descriptions for each dimension.

Subsequent term maps for each dimension were also plotted to disentangle what features characterize the behavior of each community of users (Fig 2).

Tweeters using terms in their description belonging to the academic and scientific realm (Cluster 3) stand out in the ‘Science Focus’ and ‘Science Engagement’ dimensions. In the latter dimension, users that describe themselves as opinion holders (Cluster 2) also show high values and greater ‘Social Media Activity'. This is consistent for both groups with what would be expected from the claims of their profiles. On the other hand, users who refer to their personal and private life (Cluster 1) in their description are unmistakably characterized by their high ‘Social Media Activity’. However, those who allude to their business and professional practice (Cluster 4) demonstrate the most heterogeneous behavioural pattern, even though they relatively stand out for their ‘Social Media Capital’ and ‘Science Focus'.

## Discussion and conclusions

The findings presented in this research are framed around the idea of developing a second generation of social media metrics, focused on characterizing the different social media communities of attention around science and their activities and interactions around scientific results. This work contributes to pave the way for reconciling ‘altmetric’ and bibliometric approaches in what can be seen as a broader perspective of *social media studies of science* (i.e. bringing together the interactions and network perspectives between both social media and science-related entities, activities and objects) [1, 16]. Some recent developments have also started to combine these more interactive and network perspectives. For example, the topic-actor networks based on Twitter suggested by Hellsten and Leydesdorff [42] by studying topics, tweeters, and hashtags from tweets; or the combined study of scientific author-keywords and hashtags from tweets to papers related to climate change [43], which may also be related to the broader idea of studying and characterizing different forms of “heterogeneous couplings” between social media and research objects and entities [44]. These developments support the idea of an advancement in social media metrics research towards more interactive and network perspectives, in which the distinction between those social media metrics related to research objects (primary) and those metrics related to the social media entities or users (secondary) becomes an important conceptual differentiation.

The large-scale analysis of tweeters interacting with scholarly outputs has disclosed *four dimensions of Twitter metrics around science*. These four dimensions of secondary Twitter metrics cover the frequency of tweeting, both regarding the general activity of users in the social media realm (‘Social Media Activity’) and their specific activity around science (‘Science Focus’ and ‘Science Engagement’). Regarding the latter, whereas ‘Science Engagement’ may be understood as the quantitative side of engagement by users in science, ‘Science Focus’ provides a much more nuanced insight that mirrors the overall tweeters involvement in science related issues. Finally, the number of followers, followees, and lists (‘Social Media Capital’) can be seen through the lens of social capital theory [45, 46], as these metrics basically reflect the network of relationships established by users and entail an “unceasing effort of sociability, a continuous series of exchanges in which recognition is endlessly affirmed and reaffirmed” [45] (p. 250). These four dimensions provide both a sound framework for potentially classifying users according to their behavior in the social media realm and a general benchmark for comparative analyses among users.

On the other hand, term maps based on users’ profile descriptions and the factor scores have enabled the authors to outline the type of users that stand out in each dimension. Four separated clusters of users’ terms have been found, which are aligned with previous results studying scholarly tweeters’ descriptions [32]: Tweeters who discuss their private life and are highly active (*personal description)*; tweeters who describe themselves as opinion holders and show a certain degree of engagement and activity (*opinions and own position*); tweeters who belong to the academic realm and are notable for their high science focus and engagement (*academic role*); and tweeters who refer to their professional role and show a more balanced pattern of use and relatively high social media capital (*business and practice dimension*).

### On the quest for typologies of social media users of science

Revealing the four dimensions of secondary Twitter metrics as described in this paper allows tracing the communities of users around science within the complete Twittersphere. Given the great variety of patterns and behaviors of Twitter users’ activities, the authors did not consider clustering users according to the overall range of activities and interactions as a feasible and grounded approach in the quest for a unified range of typologies. Rather, the identification of communities of users around science based on the four dimensions of metrics depicted here can be approached from a *bottom-up* perspective. For instance, one might be interested in identifying potential opinion leaders acting as influencers or science brokers (e.g. bringing science to a broader audience or to the general public). These could be operationalized as tweeters with at least medium levels of ‘Science Focus’ and well-established ‘Social Media Capital’. Science brokers would expect to devote a significant proportion of their tweeting activity to research objects, reducing the time between the publication of the paper and the first tweets, and share research articles with interesting findings. Here, there is also room for expanding the outreach of users, for instance, by distinguishing between local and global science brokers by utilizing followers’ time zone information as previously suggested by Kwak et al. [31].

Profile descriptions are also an appealing source for outlining the potential communities of users by providing hints on the background and professional activity of users. In this research, the authors have drawn on this information to complement our main empirical findings, rather than to rely primarily on the self-presentation users have chosen to provide online. Given the general scope of Twitter and the array of impressions that one user may give on Twitter, a professional role does not necessarily have to be the primary one given in the social media realm. In fact, even when a user believes they are tweeting in a professional manner, readers of their tweets may not agree [47]. Expanding our insight on scholarly identity management might be productive to build up a theory that helps to unravel the social media interactions around science. This might also have substantial implications for establishing broader social media studies of science. In this sense, there seems to be significant disciplinary differences in how researchers behave in the social media realm [48], similar to publication patterns across research domains [49, 50], that needs further investigation. This is becoming increasingly relevant as the number of researchers on Twitter is growing steadily every year [51].

### On the quest for traces of societal impact

The step towards the second generation of social media metrics fits within the paradigm change that is taking place in the larger scientometric community; impact can no longer be measured solely in scientific terms (e.g. citations), but must include other factors including societal, environmental, political, health, and economic impact [52, 53]. Therefore, moving towards examining the interaction with research objects within the full range of activities on social media platforms will help to track down potential traces of societal impact. The empirical framework of the *four dimensions of metrics around science* presented here may support other approaches for exploring the traces of societal impact of research, such as focusing on the engagement among researchers through case studies [11]. In fact, the four dimensions disclosed provide a certain benchmarking for comparative studies. Case studies and mapping may be performed for comparing users that show similar patterns on Twitter. Two users who are actually behaving similarly on Twitter may show different followers’ profiles and, therefore, have impact on different communities. In this sense, performing future examinations would be made easier if Altmetric.com shared additional Twitter metadata including the total number of tweets of the users, the date of creation of the accounts, or their number of followees.

Moreover, further conceptual discussions would also suggest the existence of *tertiary* forms of *altmetrics*; which, conceptually speaking, would refer to the combination of both *primary* and *secondary social media metrics* (in other words the combined use of metrics referring to both research objects and social media objects). A potential example of this third generation of social media metrics would be the number (or share) of tweets of a publication that come only from tweeters with high social media capital. In this example, the primary metric is the number of tweets of the publication, and the secondary metric is the number of followers used to characterize the social media capital of the tweeters.

In any case, there is still a long way ahead for the potential use of social media metrics as supporting tools in research evaluation contexts. The need for a sound definition of altmetrics based on the understanding of the processes leading to societal impact remains a crucial goal [11, 53, 54]. This must be accompanied by further empirical evidence that helps to disentangle what these metrics actually capture, since close to zero correlations have been found elsewhere between altmetrics and others indicators of societal impact [55, 56]. Besides, the current misalignment between scientometric developments and research assessment practices must be considered in the quest for social media metrics legitimacy. Jeppe, Pithan, and Heinze [57] state that the legitimacy of the most common metrics used in research evaluation is questioned and that these metrics have been proposed by outsiders (i.e., h-index) or adapted by database providers (i.e., Journal Impact Factor). Accordingly, this reflects little reputational control by the core specialists of the scientometrics field, since the emergence of new metrics and the growth of publications have not gone together with the establishment of sound intellectual foundations. This has to be understood in a context of increasing demand for standardized assessment mechanisms, which have been filled by database providers (e.g., Clarivate Analytics, Scopus). This has given these providers a powerful role in defining the standards of research quality [57]. Initiatives from the scientometric community, such as the Leiden Manifesto [58], can be seen as a response to this tension. Given the crucial role carried out by data aggregators such as Altmetric.com or Plum Analytics, similar legitimacy challenges must be faced by the community when it comes to social media metrics. In fact, given their current emerging state, this is the right moment for scientometric specialists, whether they belong to the academic (e.g. researchers) or the professional realm (e.g. consultancy, librarians), to take the lead in the quest for defining reference standards of what constitutes actual traces of societal impact and how far-reaching social media metrics can be seen as traces of them.

### Limitations

This research has some limitations which suggest avenues for future research. First, the metrics interrelated structure and, especially, the typology of users around science may differ across geographical areas, languages, and for particular kind of users (e.g. verified profiles). Second, the dataset comprises Twitter users which have posted at least one ‘tweet’ mentioning a scholarly output from August 2011. To avoid a bias representation of their Twitter activity, users with previous activity were excluded from the study. These early adopter users may perform differently on Twitter. Third, given the current emerging state of the second generation of social media metrics, new indicators are needed to expand the potential of these metrics as traces of societal impact such as those referring to the actual discussion generated on the Twitter community around scholarly outputs (e.g. number of comments received by a tweet to a paper, number of mentions to other users, etc.). Therefore, upcoming research dealing with the second generation of Twitter metrics should cross-validate the underlying structure on another sample and explore if the four dimensions of Twitter metrics around science remain stable when other metrics, languages, datasets, and geographical areas are considered.
